# Ten‐year trends of adult trauma patients in Central Denmark Region from 2010 to 2019: A retrospective cohort study

**DOI:** 10.1111/aas.14123

**Published:** 2022-08-19

**Authors:** Frederik Trier, Jesper Fjølner, Anders Høyer Sørensen, Rasmus Søndergaard, Hans Kirkegaard, Nikolaj Raaber

**Affiliations:** ^1^ Research Center for Emergency Medicine, Department of Clinical Medicine and Emergency Department Aarhus University and Aarhus University Hospital Aarhus Denmark; ^2^ Department of Emergency Medicine Randers Regional Hospital Randers Denmark; ^3^ Department of Anesthesia and Intensive Care Viborg Regional Hospital Viborg Denmark; ^4^ Research and Development, Prehospital Emergency Medical Services Central Denmark Region Aarhus Denmark

**Keywords:** age, Denmark, injury, mortality, registry, scandinavia, severe injury, trauma, trend

## Abstract

**Background:**

Trauma causes significant economic and societal burdens, and the trauma patient population and their prognosis change over time. This study aims to analyze 10‐year trends of trauma patients at a major trauma center in Central Denmark Region.

**Methods:**

Five thousand three hundred and sixty‐six patients aged ≥16 years with Injury Severity Score (ISS) > 0 admitted by trauma team activation at a major trauma center between January 1, 2010, and December 31, 2019, were included. An annual percent change with a 95% confidence interval was used to estimate trends in the mechanism of injuries. Multiple logistic regression with mortality as the outcome was adjusted for age, sex, and ISS. Admission year was used as continuous variable in logistic regressions.

**Results:**

The median age increased from 37 in 2010 to 49 in 2019, and the proportion of patients aged ≥65 doubled. The annual incidence of minor injuries (ISS 1–15) decreased from 181.3/10^5^ inhabitants in 2010 to 112.7/10^5^ in 2019. Severe injuries (ISS > 15) increased from 10.1/10^5^ inhabitants in 2010 to 13.6/10^5^ in 2019. The proportion of patients with ISS > 15 increased from 18.1% in 2010 to 31.1% in 2019. Multivariable logistic regression indicates lower 30‐day mortality for all trauma patients over the study period when adjusting for age, sex, and ISS (odds ratio: 0.94, 95% CI: 0.90–0.99). The 30‐day mortality for severely injured patients with ISS > 15 seems to decrease during the study period when adjusting for age, sex, and ISS (Odds ratio: 0.92, 95% CI: 0.87–0.97). Fall injuries increased by 4.1% annually (95% CI: 2.3%–6.1%).

**Conclusions:**

Ten‐year trends of trauma patients at a major trauma center show an increasing median age, injury severity, and number of fall injuries. The 30‐day mortality of trauma patients decreased slightly for both minor injuries and severe injuries when adjusting for age, sex, and injury severity.

**Editorial Comment**

A trauma center in Denmark reports an increase in age and injury severity of admitted patients over the last 10 years. A higher proportion of cases had falling as the injury mechanism, and there appears to have been a decrease in mortality when adjusting for patients characteristics.

## 1 INTRODUCTION

Injuries and trauma account for 8% of global mortality and significantly reduce quality of life.^1^, ^2^, ^3^, ^4^ Trauma is the leading cause of death among young people and creates a substantial economic and societal burden from subsequent disabilities and loss of potential life years.^2^, ^5^ Injuries in Scandinavia are predominantly traffic‐related, falls, and blunt trauma, with lower rates of gunshot wounds and penetrating injuries than other countries.^6^, ^7^, ^8^ In 2018, trauma patients accounted for 2405 admissions to major Danish trauma centers, of whom 6.8% died within 30 days.^3^ Common causes of death in Denmark among trauma patients have been identified as damage to the central nervous system, exsanguination and multiple organ failure.^8^, ^9^ Characterizing and analyzing trauma patients regarding epidemiology, patient demographics, interventions, and outcomes may lead to improved trauma care, identify preventable injuries and describe tendencies over time.^6^ To date, several studies have investigated long‐term trauma trends in the USA, Japan, and UK.^10^, ^11^, ^12^ Previous studies have highlighted the changes in the mechanism of injury, mortality and demographics; particularly, a rise in fall injuries and increasing age were among the compelling findings.^11^, ^12^ Moreover, trauma mortality is changing due to demographic alterations and the increasing proportion of elderly trauma patients with higher mortality.^6^, ^13^ In recent years, the impact of long‐term changes in the Danish trauma population has not been explored. Trauma registries can facilitate analysis, injury prevention, comparison and benchmarking across regions and countries.^6^ Hence, this study's aim is to analyze 10‐year trends of trauma patients admitted to the major trauma center in Central Denmark Region focusing on annual incidence, severity, mortality, and the mechanism of injury (MOI). We will further investigate the subgroup of patients with severe injuries (Injury Severity Score [ISS] > 15).

## 2 METHODS

This is a 10‐year (2010–2019) retrospective cohort study conducted at a major trauma center in Denmark. The study followed the STROBE guidelines.^14^

### 2.1 Setting and data collection

Our Trauma Registry (TR) is the trauma registry of Aarhus University Hospital Trauma Center (AUH‐TC). AUH‐TC is a tertiary trauma center with highly specialized surgery and critical care. The catchment area for minor injuries covers the municipality (population 350,000) and major trauma patients from the region (population 1.3 million). The population count, mean age, life expectancy in Central Denmark region was collected retrospectively at Statistics Denmark.^4^ The annual number of trauma team activations (TTA) at AUH‐TC is approximately 600, and over 150 of these patients are severely injured (ISS > 15).^3^ AUH‐TC is the referral trauma center for four regional acute‐care hospitals in Central Denmark Region. Minor injuries without TTA treated in the Emergency Department (ED) and at acute‐care hospitals are excluded from TR. The pre‐hospital emergency medical service in Central Denmark Region consists of 69 ambulances (staffed by paramedics or emergency medical technicians) and 10 rapid response vehicles (staffed by a paramedic and an anesthesiologist). No formal change in the catchment occurred, but the national helicopter emergency medical service (HEMS) was implemented in 2014 and comprises four helicopters staffed with paramedics and an anesthesiologist. The pre‐hospital unit requests TTA following predefined criteria (Table S1). TTA involves a minimum of 10 professionals, including an anesthesiologist, orthopedic surgeon, emergency physician, nurse anesthetist, trauma nurse, medical laboratory technologist, radiographer, medical secretary, supporting staff, and physicians or surgeons from relevant medical specialties. TR data is collected by hospital administrative staff during the patient's treatment. Data in TR include patient demographics, injury description, timeline, ISS, and MOI. MOI is categorized by road traffic collision, scooter, motorcycle, bicycle, fall, horse, pedestrian, violence, stabbing, self‐harm, gunshot, and other mechanisms, such as animal‐related injuries, crushing, struck by objects, machinery accidents, sports injuries or other miscellaneous injuries. Fall injuries include falls from all heights.

### 2.2 Outcome

Danish trauma centers use the Abbreviated Injury Scale (AIS) to score each injury sustained by trauma patients.^15^, ^16^ The ISS is calculated based on the assigned AIS scores done by AIS‐certified physicians and medical students. To avoid interpersonal discrepancies between AIS scores assigned, the coding team regularly discussed cases and reviewed the process of assigning codes to assure consistency. Severe injuries were defined as ISS > 15. Data for 30‐day mortality were retrieved from patients' medical records using the Danish Civil Registration number (CRS). The Danish Civil Registration System contains a vital status for all Danish citizens and non‐citizens who reside in Denmark.^17^

### 2.3 Patients

**FIGURE 1:**
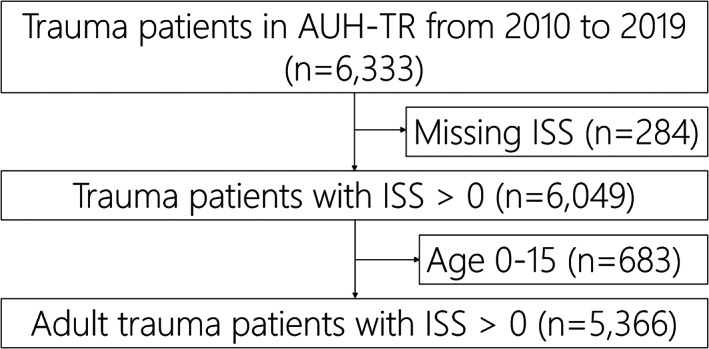
Patient flow chart for inclusion and exclusion criteria. Non‐citizens with incomplete 30‐day follow‐up (*n* = 113) are excluded from mortality analysis. Excluded patients with missing ISS (*n* = 284) were due to incomplete medical record, death before arrival, duplicate entries, cancelation of trauma team activation, cardiac arrest or other medical conditions. AUH‐TR, Aarhus University Hospital Trauma Registry; ISS, Injury Severity Score

Trauma patients admitted with TTA to AUH‐TC with an ISS > 0 and aged ≥16 were included in the analyses. Figure 1 shows patient inclusion and exclusion for the study. Patients with major trauma initially admitted to an acute‐care hospital with secondary transfer to AUH‐TC within 24 h were also included.

### 2.4 Statistics

Descriptive data were reported as numbers and percentages. Continuous data were reported as mean and standard deviation for normally distributed data, and median and interquartile range (IQR) for non‐normal distributed data. Nonparametric comparisons over time for ISS and age were performed using the Kruskal–Wallis test. The annual incidence rates were calculated using the catchment area population count separately for the municipality (ISS 1–15) and Central Denmark Region (ISS > 15).^4^ An average linear trend in the change in sex, ISS > 15 and MOI proportions during the 10‐year study period was calculated using logistic regression with year as a continuous independent variable. This computed the estimated annual percent change (APC) with a 95% confidence interval. Logistic regression with 30‐day mortality as outcome adjusted for age, sex, and ISS were calculated and admission year was used as continuous independent variable. STATA version STATA/IC 17.0 (StataCorp. 2019, Texas) was used for all statistical analyses. A two‐sided *p* value of <.05 was considered statistically significant.

### 2.5 Ethics and approvals

This study was a registry study and exempted from trial registration following Danish law due to the data anonymity. The hospital board of directors approved the study.

## 3 RESULTS

**FIGURE 2:**
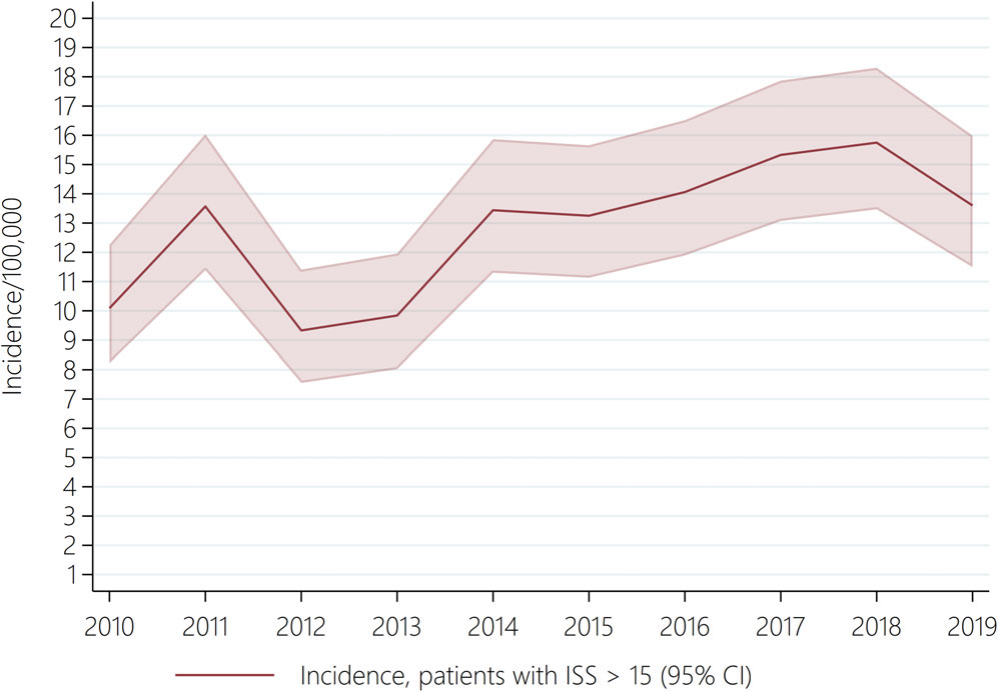
Annual incidence of trauma patients admitted by trauma team activation to AUH‐TC with Injury Severity Score (ISS) > 15 per 100,000 inhabitants from 2010 to 2019. CI, confidence interval

**FIGURE 3:**
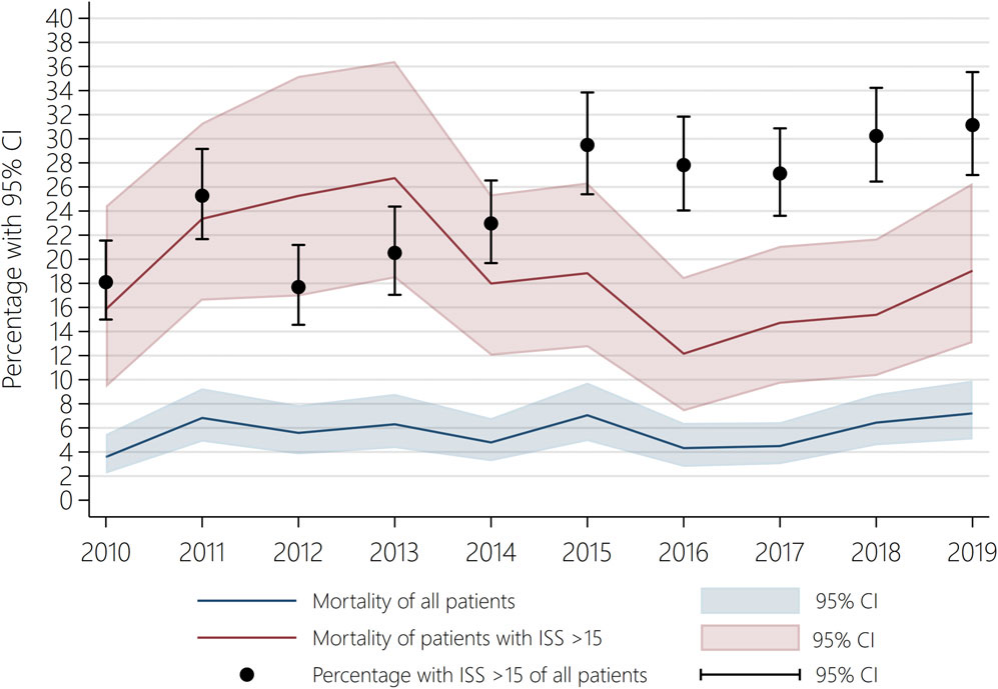
Temporal trend in 30‐day mortality of all patients and 30‐day mortality of patients with Injury Severity Score (ISS) > 15 from 2010 to 2019. The percentage of trauma patients with ISS > 15 is shown in black from 2010 to 2019. CI, confidence interval

During the study, 5366 adult trauma patients aged ≥16 with ISS > 0 between January 1, 2010 and December 31, 2019 were included (Figure 1). Table 1 shows the patient characteristics and outcome. The median age increased significantly from 37 years in 2010 to 49 years in 2019 (Table 1). The proportion of patients aged ≥65 increased significantly from 11.7% (*n* = 65) in 2010 to 23.5% (*n* = 111) in 2019 (Table 1). The annual incidence of minor injuries (ISS 1–15) with TTA decreased from 181.3/10^5^ inhabitants (95% CI: 165.1–198.7) in 2010 to 112.7/10^5^ (95% CI: 100.8–125.6) in 2019 (Table S3). The annual incidence of severe injuries (ISS > 15) increased from 10.1/10^5^ (95% CI: 8.2–12.3) in 2010 to 13.6/10^5^ (95% CI: 11.5–16.0) in 2019 (Figure 2, Table S3). The severity of the trauma patient population at AUH‐TC changed, as the proportion of patients with ISS > 15 increased significantly from 18.1% in 2010 to 31.1% in 2019 (Table 1, Figure 3, Figure S1).

**TABLE 1:** Patient characteristics and outcomes from 2010 to 2019

Year	2010	2011	2012	2013	2014	2015	2016	2017	2018	2019	Statistics
Patients, *n*	558	542	537	492	605	468	532	601	559	472	
Male, *n* (%)	375 (67.2)	373 (69.0)	352 (65.7)	334 (68.0)	392 (65.3)	298 (65.2)	355 (67.0)	398 (66.8)	408 (73.0)	319 (67.7)	0.3 (−0.3 to 1.0)^a^
Age, median (IQR)	37 (24 to 53)	39 (25 to 52.5)	38 (25 to 54)	42 (26 to 57)	41 (25 to 55)	42 (26 to 57)	43 (25 to 59)	41 (25 to 58)	45 (27 to 61)	49 (27 to 63)	<0.01^b^
Age, *n* 16–64 (%)	490 (88.3)	461 (85.4)	460 (86.0)	419 (85.3)	518 (86.2)	390 (83.9)	430 (81.0)	483 (80.8)	433 (77.5)	361 (76.5)	
Age, *n* ≥65 (%)	65 (11.7)	79 (14.6)	75 (14.0)	72 (14.7)	83 (13.8)	75 (16.1)	101 (19.0)	115 (19.2)	126 (22.5)	111 (23.5)	
30‐day mortality age 16–64, *n* (%)	10 (2.1)	15 (3.4)	18 (4.1)	20 (4.8)	18 (3.5)	18 (4.7)	9 (2.1)	8 (1.7)	18 (4.3)	12 (3.4)	
30‐day mortality age ≥ 65, *n* (%)	10 (15.4)	22 (28.2)	11 (15.1)	11 (15.3)	10 (12.2)	15 (20.0)	14 (14.0)	19 (16.7)	18 (14.8)	22 (20.0)	

*Note*: Missing Danish Civil Registration number in 2010 (*n* = 4), 2011 (*n* = 16), 2012 (*n* = 19), 2013 (*n* = 4), 2014 (*n* = 11), 2015 (*n* = 12), 2016 (*n* = 6), 2017 (*n* = 12), 2018 (*n* = 18), 2019 (*n* = 11).

Abbreviation: ISS, Injury Severity Score.

^a^ Annual percent change.

^b^ Kruskal–Wallis test.

### 3.1 Mortality

**FIGURE 4:**
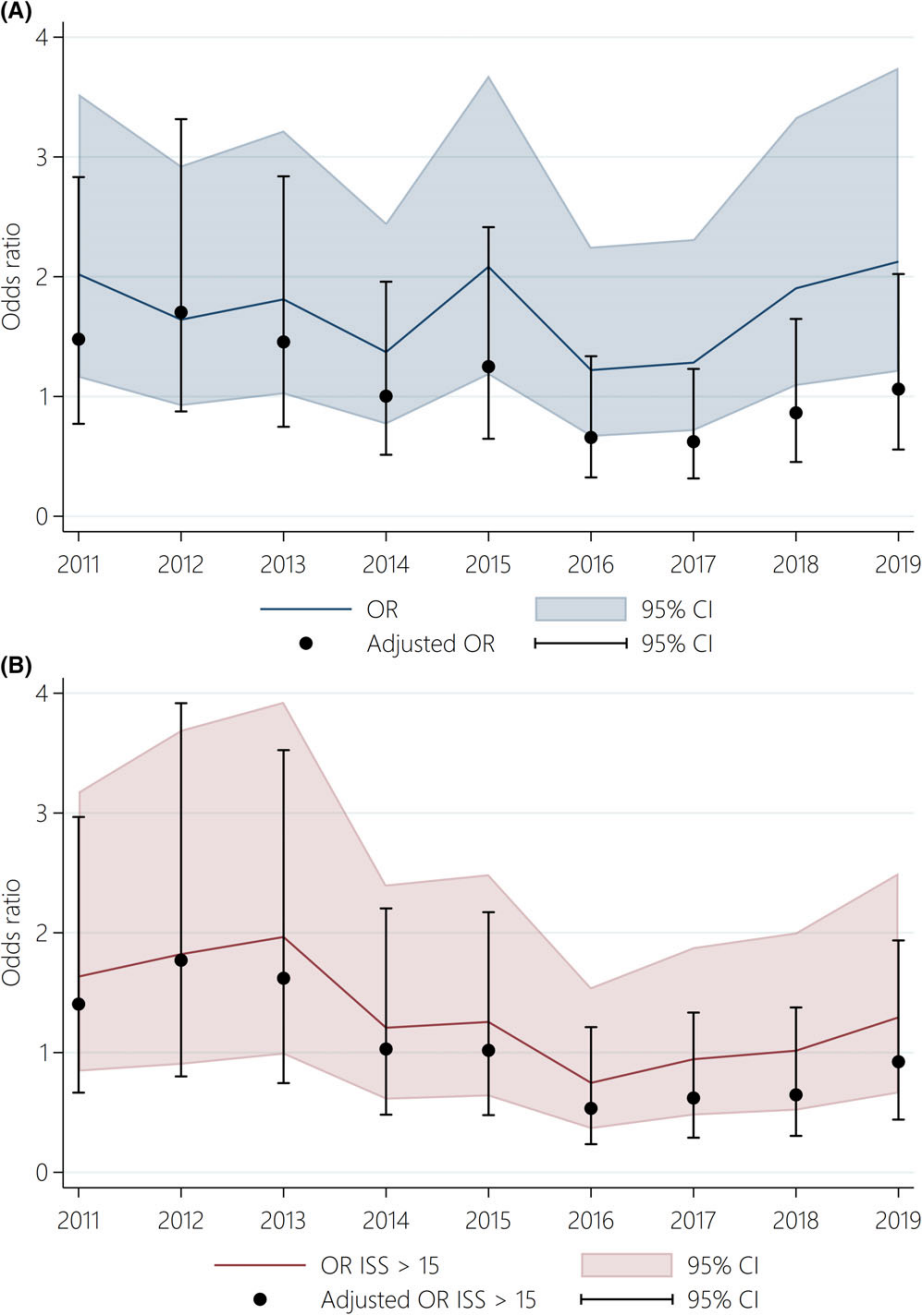
(A) Odds ratio (OR) for 30‐day mortality of all trauma patients with 95% confidence interval (CI) from 2011 to 2019. Year 2010 was used as reference and year was used as a continuous variable. The adjusted OR shown in black was adjusted for age, sex, and Injury Severity Score. (B) Odds ratio (OR) for 30‐day mortality of severely injured trauma patients (Injury Severity Score [ISS] > 15) with 95% confidence interval (CI) from 2011 to 2019. Year 2010 was used as reference and year was used as a continuous variable. The adjusted OR shown in black was adjusted for age, sex, and ISS

The 30‐day mortality of all patients were 3.6% in 2010 and 7.4% in 2019 (Table 1, Figure 3). The 30‐day mortality of patients with ISS > 15 were 15.8% in 2010 and 19.6% in 2019 (Table 1, Figure 3). In multivariate regression analyses (Table 2, Table S4, Figure 4A,B), the 30‐day mortality for all patients decreased from 2010 to 2019 when adjusting for age, sex, and ISS (odds ratio: 0.94, 95% CI: 0.90–0.99). Additionally, the 30‐day mortality for patients with ISS > 15 decreased from 2010 to 2019 when adjusting for age, sex, and ISS (odds ratio: 0.92, 95% CI: 0.87–0.97). The odds ratio for 30‐day mortality was 5.93 for patients aged ≥65 (95% CI: 4.66–7.54) compared to patients aged 16–64.

**TABLE 2:** Multivariable logistic regression analysis for 30‐day mortality of trauma patients

	All patients, *n* = 5253		Patients with ISS 1–15, *n* = 3941		Patients with ISS > 15, *n* = 1312	
	OR (95% CI)	*p* value	OR (95% CI)	*p* value	OR (95% CI)	*p* value
Age	1.05 (1.04 to 1.06)	<.01	1.06 (1.05 to 1.08)	<.01	1.03 (1.03 to 1.04)	<.01
16–64	Reference		Reference		Reference	
≥65	5.93 (4.66 to 7.54)	<.01	8.35 (4.82 to 14.46)	<.01	3.33 (2.49 to 4.44)	<.01
Sex, male	1.05 (0.82 to 1.36)	.683	1.03 (0.58 to 1.82)	.930	0.78 (0.58 to 1.05)	.106
ISS	1.11 (1.10 to 1.12)	<.01	1.11 (1.05 to 1.18)	<.01	1.07 (1.06 to 1.09)	<.01
Year (2010–2019)	1.02 (0.98 to 1.07)	.267	1.08 (0.98 to 1.18)	.129	0.95 (0.91 to 1.00)	.059
Year adjusted for age, sex and ISS	0.94 (0.90 to 0.99)	.012	1.01 (0.92 to 1.12)	.778	0.92 (0.87 to 0.97)	.002

*Note*: Odds ratios are calculated for all patients, patients with ISS 1–15 and patients with ISS > 15 separately. Admission year was used as a continuous variable. Missing Civil Registration number in 2010 (*n* = 4), 2011 (*n* = 16), 2012 (*n* = 19), 2013 (*n* = 4), 2014 (*n* = 11), 2015 (*n* = 12), 2016 (*n* = 6), 2017 (*n* = 12), 2018 (*n* = 18), 2019 (*n* = 11).

Abbreviations: CI, confidence interval; ISS, Injury Severity Score; OR, odds ratio.

### 3.2 Mechanism of injury

Proportions of MOI changed during the 10‐year study period. The 95% CI indicates significant increases in the annual percent change (APC) in fall injuries of 4.1% (95% CI: 2.3%–6.1%). Contrary, we found significant APC decreases in pedestrian injuries by −4.7% (95% CI: −8.6% to −0.7%) and violence −14.0 (95% CI: −18.7% to −8.9%) (Table S2).

### 3.3 Transport

Transport and admission data were available from 2015 to 2019. The proportion of trauma patients transferred from other hospitals decreased numerically from 53 (11.3%) in 2015 to 36 (7.6%) in 2019 (Table S5). The proportion of patients admitted directly from surrounding municipalities increased numerically from 165 (43.4%) in 2015 to 233 (51.2%) in 2019. The proportion of patients with ISS > 15 admitted directly from surrounding municipalities was 70 (65.4%) in 2015 and increased numerically to 110 (78.0%) in 2019. The proportion of patients admitted by HEMS increased numerically from 48 (10.3%) in 2015 to 63 (13.4%) in 2019.

## 4 DISCUSSION

In this study, we investigated 10‐year trends in demographics, injury severity, 30‐day mortality, and MOI among trauma patients admitted to AUH‐TC. We found an increase in the median age, and the proportion of patients aged ≥65 doubled. The annual incidence and proportion of severely injured patients (ISS > 15) increased throughout the study period. The crude 30‐day mortality of all patients including those severely injured did not change despite an increased injury severity but did, however, decrease marginally when adjusting for age, sex, and ISS. Long‐term trends in the mechanism of injuries displayed a significant increase in fall injuries.

### 4.1 Changing demographics

We found that the median age of adult trauma patients at AUH‐TC peaked at 49 years in 2019. Furthermore, we found an increase in the proportion of patients aged ≥65. In a previous study from AUH‐TC, the median age of all trauma patients increased from 29 to 32 between 2000 and 2008.^18^ The mean age and the life expectancy increased from 39.5 to 41.4 and 80.2 to 82.1 in Central Denmark Region during the 10‐year study period, respectively.^4^ These results reveal accelerated aging of the trauma patient population at AUH‐TC. The aging of the trauma patient population parallels with many previous trauma studies worldwide.^10^, ^11^, ^12^ Kehoe et al.^12^ studied major trauma from 1990 to 2013 in the UK and described how the mean age of major trauma patients increased from 36.1 to 53.8, and the proportion of patients aged ≥75 increased from 8.1% to 26.9%. Pre‐existing medical conditions such as diabetes mellitus, obesity, heart disease and hypertension are more frequent and contribute to morbidity in elderly trauma patients.^19^ In Norway, Cuecas‐Østrem et al. studied geriatric trauma patients nationwide from 2015 to 2018.^20^ They included adult trauma patients with a New Injury Severity Score (NISS) ≥ 9 and older than 16 years and compared them to patients aged ≥65. Geriatric trauma patients in that study had a 30‐day mortality of 13.6% compared to 2.6% of patients aged from 16 to 64 years. Another study from Germany found increased mortality independent of ISS from age ≥ 56 in severely injured elderly trauma patients.^21^ A nationwide study of death causes in Sweden reported a decline in injury‐related mortality among working‐age but an increase among elderly patients.^13^ Several studies highlighted the frailty and excess mortality of elderly trauma patients and the need for increased focus on this age group.^13^, ^22^ The aging population and possibly more active elders results in a changing trauma patient population.

### 4.2 Injury severity

We found an increasing injury severity, and the proportion of severely injured patients with ISS > 15 climbed from 18.1% in 2010 to a maximum of 31.1% in 2019. The median ISS of severely injured patients (ISS > 15) was 24 in 2019 at AUH‐TC. The proportion of patients with ISS > 15 was 33.9% at a major trauma center in Norway and 21.1% at a major trauma center in Sweden from 2009 to 2011.^23^ When compared with other major trauma centers, the median ISS of trauma patients with ISS > 15 were 17 in Spain, 24 in Hong Kong, and between 22 and 25 in TraumaRegister DGU® (TR‐DGU).^24^, ^25^

### 4.3 Mortality

In 2019, we observed a 7.4% crude 30‐day mortality of patients aged ≥16 years with ISS > 0. We identified a survival benefit between 2010 and 2019 when adjusting for age, sex, and ISS. We calculated an odds ratio for 30‐day mortality of 5.93 among patients aged ≥65 compared to patients aged 16–64. When focusing on Central Denmark Region, Mikkelsen et al. studied trauma patients admitted to AUH‐TC from 2000 to 2011 and found a 4.4% mortality and no improved survival during the study period.^26^ However, they did not specify ISS inclusion or exclusion criteria for patients with missing CRS.^26^ In a Scandinavian study of trauma patients with ISS > 0 and older than 15 years they observed 30‐day mortality of 5.6% in Norway and 3.2% in Sweden.^23^ Also, a Swedish study from 2013 to 2017 found a 4.6% 30‐day mortality among trauma patients with ISS > 0 of all ages treated at major trauma centers.^27^ In a Japanese nationwide trauma registry study from 2004 to 2013 the adjusted in‐hospital mortality of adult trauma patients with ISS >3 decreased annually during the study period.^11^ For severe injuries, we found a 19.6% crude 30‐day mortality of patients aged ≥16 years with ISS > 15. Several European studies^7^, ^24^, ^28^ and a study from Hong Kong^25^ revealed a 30‐day mortality of trauma patients with ISS > 15 varying between 14.9% and 22.4%. In Japan, the in‐hospital mortality of adult trauma patients with ISS ≥ 16 decreased from 28.5% in 2004 to 15.7% in 2013.^11^ The 30‐day mortality for patients with ISS > 15 varies between major trauma centers but seems comparable to the results in the present study.

### 4.4 Mechanism of injury

We found a significant APC increase in fall injuries of 4.1%. We found a decreasing APC in pedestrian injuries of −4.7% and scooter injuries of −6.0%. Many studies have also reported increasing numbers of falls, especially in patients aged ≥65.^10^, ^11^, ^12^, ^20^ In the UK, the proportion of fall injuries <2 m among severely injured patients increased from 4.7% in 1990 to 39.1% in 2013.^12^ Ten‐year trends from 2005 to 2014 in a statewide trauma registry in Oklahoma, USA, found the APC of fall injuries to increase by 4.0% (95% CI: 3.1%–4.9%), and the number of fall injuries among patients ≥65 more than doubled. Furthermore, the APC of pedestrian injuries decreased by −1.5% during the 10‐year study period in that trauma patient population.^10^ We found APC increases in stabbings of 6.8% and gunshots of 15.0% but decreases in violence of −14.0%. Twenty‐year trends of trauma patients in the UK found the proportion of both gunshot and stabbing to increase from 0.2% in 1990 to 1.9% in 2013.^12^

### 4.5 Centralization of trauma care

In this study, a greater number of severely injured patients were admitted directly from surrounding municipalities and fewer patients were transferred from 2015 to 2019. Together with less transferred patients the increasing injury severity and proportion of patients with ISS > 15 during the study suggests that more severely injured trauma patients are admitted directly to AUH‐TC from other parts of Central Denmark Region. The growth in proportions of severe injuries at AUH‐TC could be explained by more focus on centralized trauma care, with severely injured patients transported directly to the highest level of expertise at the major trauma center. Furthermore, the number of rapid response vehicles staffed with anesthesiologists and paramedics increased during the observed period and the national HEMS was introduced in 2014 and both contributing to the change in referral pattern. Prior to our results, Brink et al. in 2008 found that the percentage of patients transferred to AUH‐TC increased from 3.9% to 13.4%.^18^ In a systematic review and meta‐analysis from 2018, Sewalt et al. found a modest association between high volume centers and improved survival in severely injured trauma patients.^29^ A Japanese study found reduced in‐hospital mortality of severe trauma patients in high volume trauma centers (≥100 annual patients with ISS >15) compared to low volume trauma centers (1–49 annual patients with ISS >15).^30^ However, in a recent risk‐adjusted mortality analysis study in Sweden by Strömmer et al., the survival benefit of major trauma centers diminished in severely injured (NISS > 15) adult trauma patients and the only difference in mortality were due to traumatic brain injuries.^31^ The centralization of trauma care may improve survival but the effect of centralization remains unclear.

### 4.6 Strengths and limitations

We included long‐term trends in mortality. The percentage of missing patients is low, and most major trauma patients in Central Denmark Region (22% of the Danish population) are admitted to AUH‐TC.^32^ Our study, however, has potential limitations that affect the interpretation. We found significant changes in MOI, but the number of patients in each category of MOI was limited and we did not include minor injuries treated in the ED, thus complicating the identification of long‐term trends in MOI. The level of undertriage in our region is unknown. Studies have shown inter‐rater discrepancies in AIS scores; however, these discrepancies rarely affect the ISS.^33^ Although the TTA criteria remained the same, the emergency physician's threshold to activate the trauma team could have changed during the study period. The implementation of national HEMS in 2014 and centralization of trauma care may have changed the referral pattern. In 2018, Weile et al.^34^ found a heterogeneous composition of the trauma team and TTA criteria and poor registration practice at regional trauma facilities in Denmark and some unknown number of major trauma patients may still be treated at acute‐care hospitals in Central Denmark Region. Therefore, a national benchmark study including the entire Danish trauma patient population is warranted.

## 5 CONCLUSION

In this single‐center 10‐year retrospective cohort study, we observed increasing injury severity and proportion of elderly but unchanged crude 30‐day mortality. The annual incidence of minor injuries with TTA decreased contrary to the rise in the annual incidence of severe injuries. Age, sex, and injury severity‐adjusted survival improved modestly from 2010 to 2019. Fall injuries are significantly increasing, and trauma team activations with patients aged ≥65 doubled from 2010 to 2019.

## AUTHOR CONTRIBUTIONS

Frederik Trier, Nikolaj Raaber, Hans Kirkegaard, Jesper Fjølner, Anders Høyer Sørensen and Rasmus Søndergaard designed this study. Frederik Trier, Nikolaj Raaber, Hans Kirkegaard and Jesper Fjølner contributed to data collection. Frederik Trier, Nikolaj Raaber, Hans Kirkegaard and Jesper Fjølner performed the data analysis and interpretation of data. Frederik Trier wrote the manuscript. Hans Kirkegaard, Jesper Fjølner, Nikolaj Raaber, Rasmus Søndergaard and Anders Høyer Sørensen critically revised the manuscript. All authors approved the final manuscript.

## FUNDING INFORMATION

Department funding only.

## CONFLICT OF INTEREST

Nothing to declare.

## Supporting information


**Figure S1** Number of trauma patients admitted by trauma team activation to AUH‐TC within four categories of Injury Severity Score (ISS) categories from 2010 to 2019.

**Table S1** Trauma team is activated by a score of 2 ≥points based on the clinical characteristics, physiological parameters, mechanism of injury, comorbidity or age.
**Table S2** Trends in mechanism of injury from 2010 to 2019. Admission year was used as a continuous variable.
**Table S3** Annual number of patients (*n*) and annual incidence per 100,000 inhabitants. The catchment area for minor injuries (ISS 1–15) and severe injuries (ISS > 15) is Aarhus Municipality and Central Denmark Region, respectively. Population counts for inhabitants aged ≥16 were included.
**Table S4** Multivariable logistic regression analysis for 30‐day mortality of trauma patients. Year 2010 was used as a reference and admission year was used as a continuous variable.
**Table S5** Catchment area and admission type for trauma patients from 2015 to 2019.

## Data Availability

The data that support the findings of this study are available on request from the hospitals board of directors at Aarhus University Hospital. The data are not publicly available.
