# Supplementary figures and images for: Ten‐year trends of adult trauma patients in Central Denmark Region from 2010 to 2019: A retrospective cohort study
Source: Acta Anaesthesiol Scand. 2022 Aug 19;66(9):1130–7. doi: 10.1111/aas.14123 (PMC9541060; doi:10.1111/aas.14123)

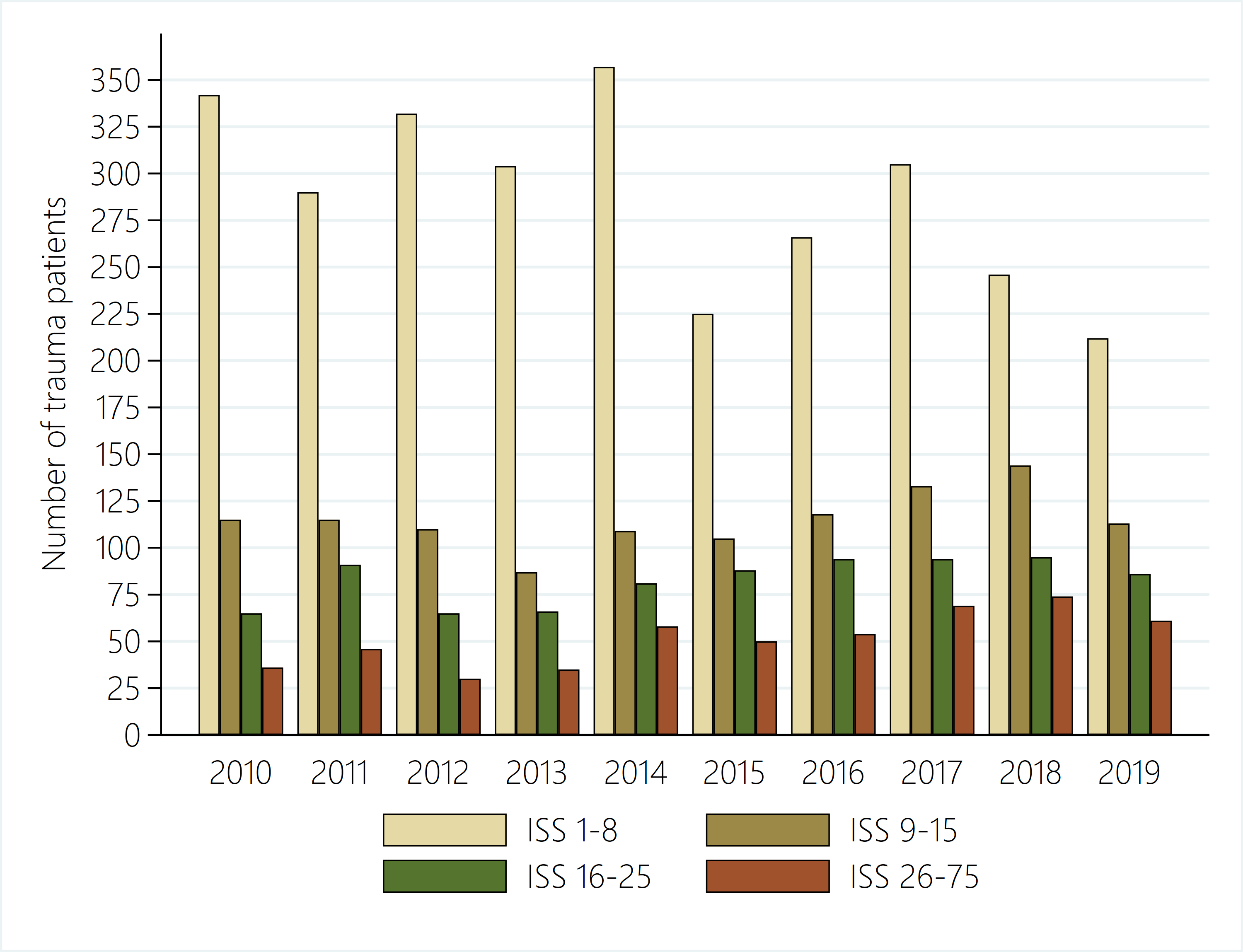

Supplement: Supplementary file 1 — Figure S1 Number of trauma patients admitted by trauma team activation to AUH‐TC within four categories of Injury Severity Score (ISS) categories from 2010 to 2019. [file AAS-66-1130-s002.png]
