# Supplementary material for: Ten‐year trends of adult trauma patients in Central Denmark Region from 2010 to 2019: A retrospective cohort study
Source: Acta Anaesthesiol Scand. 2022 Aug 19;66(9):1130–7. doi: 10.1111/aas.14123 (PMC9541060; doi:10.1111/aas.14123)
Supplement: Supplementary file 2 — Table S1 Trauma team is activated by a score of 2 ≥points based on the clinical characteristics, physiological parameters, mechanism of injury, comorbidity or age. Table S2 Trends in mechanism of injury from 2010 to 2019. Admission year was used as a continuous variable. Table S3 Annual number of patients (n) and annual incidence per 100,000 inhabitants. The catchment area for minor injuries (ISS 1–15) and severe injuries (ISS > 15) is Aarhus Municipality and Central Denmark Region, respectively. Population counts for inhabitants aged ≥16 were included. Table S4 Multivariable logistic regression analysis for 30‐day mortality of trauma patients. Year 2010 was used as a reference and admission year was used as a continuous variable. Table S5 Catchment area and admission type for trauma patients from 2015 to 2019. [file AAS-66-1130-s001.docx]

**Supplementary**

Table S1: Trauma team is activated by a score of 2 ≥ points based on the clinical characteristics, physiological parameters, mechanism of injury, comorbidity or age.

|  | 0 | 1 | 2 | Point |
| --- | --- | --- | --- | --- |
| Consciousness | Responsive = GCS 15. | Partial response GCS 13–14.  Brief unconscious. | Completely unresponsive GCS < 13. |  |
| Breathing | Normal. | Dyspnea. | Saturation < 90.  Frequency < 10 or > 30. |  |
| Circulation | Systolic BP > 90 mmHg. | Cold, sweating, pulse > 100. | Systolic BP < 90 mmHg. |  |
| Chest | No tenderness/light tenderness. | Persistent pain, +/− touch. | Open lesion. |  |
| Stomach | No tenderness/light tenderness. | Persistent pain. | Open lesion. |  |
| Neck/spine | No tenderness/light tenderness. | Constant or persistent pain. Tingling in arms or legs. | Paralysis, fracture, or suspected spine fractures. |  |
| Mechanism of injury | Low energy. | Pedestrian/bicycle or high energy. | Burns (second or third degree):  Children > 10%  Adults > 15%  Penetrating injury in head/neck. |  |
| Increased risk: Age and/or comorbidity |  | < 6 years  > 75 years  Comorbidity |  |  |
| CGS = Glasgow Coma Scale. BP = Blood pressure | | | | |

Table S2: Trends in mechanism of injury from 2010-2019. Admission year was used as a continuous variable.

|  | 2010,  n (%) | 2011,  n (%) | 2012,  n (%) | 2013,  n (%) | 2014,  n (%) | 2015,  n (%) | 2016,  n (%) | 2017,  n (%) | 2018,  n (%) | 2019,  n (%) | APC ^a^  (95% CI) |
| --- | --- | --- | --- | --- | --- | --- | --- | --- | --- | --- | --- |
| Road traffic collision | 181 (37.8) | 157 (30.5) | 171 (33.0) | 118 (24.6) | 167 (27.6) | 151 (32.6) | 191 (35.9) | 189 (31.5) | 167 (29.9) | 143 (30.3) | −0.7 (−2.1 to 0.7) |
| Motorcycle | 23 (4.8) | 33 (6.4) | 19 (3.7) | 29 (6.1) | 41 (6.8) | 23 (4.9) | 31 (5.8) | 27 (4.5) | 35 (6.3) | 27 (5.7) | −0.9 (−3.0 to 5.0) |
| Scooter | 32 (6.7) | 23 (4.5) | 23 (4.4) | 26 (5.4) | 29 (4.8) | 27 (5.8) | 24 (4.5) | 28 (4.7) | 13 (2.3) | 15 (3.2) | −6.0 (−10.0 to −1.8) |
| Bicycle | 50 (10.4) | 71 (13.8) | 77 (14.8) | 93 (19.4) | 117 (19.3) | 61 (13.0) | 59 (11.1) | 81 (13.5) | 75 (13.4) | 79 (16.7) | 0.4 (−1.9 to 2.8) |
| Fall | 86 (18.0) | 103 (20.0) | 97 (18.7) | 90 (18.8) | 126 (20.8) | 94 (20.1) | 126 (23.7) | 139 (23.1) | 150 (26.8) | 116 (24.) | 4.1 (2.3 to 6.1) |
| Horse | 12 (2.5) | 20 (3.9) | 22 (4.2) | 23 (4.8) | 21 (3.5) | 14 (3.0) | 12 (2.3) | 24 (4.0) | 17 (3.0) | 12 (2.5) | −2.6 (−7.4 to 2.5) |
| Pedestrian | 23 (4.8) | 27 (5.3) | 45 (8.7) | 27 (5.6) | 30 (5.0) | 15 (3.2) | 27 (5.1) | 32 (5.3) | 28 (5.0) | 12 (2.5) | −4.7 (−8.6 to −0.7) |
| Violence | 31 (6.5) | 29 (5.6) | 8 (1.5) | 18 (3.8) | 15 (2.5) | 15 (3.2) | 16 (3.0) | 12 (2.0) | 9 (1.6) | 6 (1.3) | −14.0 (−18.7 to −8.9) |
| Stabbing | 11 (2.3) | 15 (2.9) | 14 (2.7) | 18 (3.8) | 14 (2.3) | 18 (3.9) | 14 (2.6) | 24 (4.0) | 24 (4.3) | 22 (4.7) | 6.8 (1.4 to 12.5) |
| Gunshot | 4 (0.8) | 1 (0.2) | 1 (0.2) | 1 (0.2) | 2 (0.3) | 4 (0.9) | 2 (0.4) | 6 (1.0) | 3 (0.5) | 7 (1.5) | 15.0 (0.9 to 31.1) |
| Self-harm | 8 (1.7) | 7 (1.4) | 6 (1.2) | 8 (1.7) | 7 (1.2) | 13 (2.8) | 6 (1.1) | 9 (1.5) | 7 (1.3) | 7 (1.5) | −0.3 (−7.8 to 7.7) |
| Other mechanism ^b^ | 18 (3.8) | 28 (5.5) | 36 (6.9) | 28 (5.9) | 36 (6.0) | 33 (7.1) | 24 (4.5) | 30 (5.0) | 31 (5.6) | 27 (5.7) | 0.0 (−3.5 to 4.4) |
| ^a^ Annual percent change. ^b^ Other mechanism of injuries includes crushing, struck by object or sports injuries. Mechanism of injury were missing in 2010 (n = 79), 2011 (n = 28), 2012 (n = 18), 2013 (n = 13). | | | | | | | | | | | |

Table S3: Annual number of patients (n) and annual incidence per 100,000 inhabitants. The catchment area for minor injuries (ISS 1–15) and severe injuries (ISS > 15) is Aarhus Municipality and Central Denmark Region, respectively. Population counts for inhabitants aged ≥ 16 were included.

| Year | Patients ISS 1–15, n | Population in Aarhus Municipality | Incidence, patients with ISS 1–15 (95% CI) | Patients ISS > 15, n | Population in Central Denmark Region | Incidence, patients with ISS > 15 (95% CI) |
| --- | --- | --- | --- | --- | --- | --- |
| 2010 | 457 | 252,034 | 181.3 (165.1 to 198.7) | 101 | 1,001,230 | 10.1 (8.2 to 12.3) |
| 2011 | 405 | 256,209 | 158.1 (143.1 to 174.2) | 137 | 1,009,841 | 13.6 (11.4 to 16.0) |
| 2012 | 442 | 259,941 | 170.0 (154.6 to 186.6) | 95 | 1,018,037 | 9.3 (7.6 to 11.4) |
| 2013 | 391 | 264,493 | 147.8 (133.6 to 163.2) | 101 | 1,026,349 | 9.8 (8.0 to 12.0) |
| 2014 | 466 | 269,202 | 173.1 (157.8 to 189.5) | 139 | 1,034,105 | 13.4 (11.3 to 15.9) |
| 2015 | 330 | 271,306 | 121.6 (108.9 to 135.5) | 138 | 1,041,387 | 13.3 (11.1 to 15.7) |
| 2016 | 384 | 275,133 | 139.6 (126.0 to 154.2) | 148 | 1,052,315 | 14.1 (11.9 to 16.5) |
| 2017 | 438 | 279,532 | 156.7 (142.4 to 172.1) | 163 | 1,063,275 | 15.3 (13.1 to 17.9) |
| 2018 | 390 | 284,042 | 137.3 (124.0 to 151.6) | 169 | 1,072,986 | 15.8 (13.5 to 18.3) |
| 2019 | 325 | 288,446 | 112.7 (100.8 to 125.6) | 147 | 1,080,529 | 13.6 (11.5 to 16.0) |
| ISS = Injury Severity Score. CI = Confidence Interval. | | | | | | |

Table S4: Multivariable logistic regression analysis for 30-day mortality of trauma patients. Year 2010 was used as a reference and admission year was used as a continuous variable.

|  | All patients, n = 5253 | | Patients with ISS > 15, n = 1312 | |
| --- | --- | --- | --- | --- |
| Year | OR (95% CI) | p-value | OR (95% CI) | p-value |
| 2010 | Reference |  | Reference |  |
| 2011 | 2.02 (1.16 to 3.53) | 0.013 | 1.63 (0.84 to 3.18) | 0.148 |
| 2012 | 1.64 (0.92 to 2.93) | 0.093 | 1.82 (0.90 to 3.69) | 0.097 |
| 2013 | 1.81 (1.02 to 3.22) | 0.043 | 1.96 (0.98 to 3.93) | 0.056 |
| 2014 | 1.37 (0.77 to 2.45) | 0.288 | 1.21 (0.61 to 2.30) | 0.592 |
| 2015 | 2.08 (1.18 to 3.68) | 0.012 | 1.26 (0.63 to 2.49) | 0.514 |
| 2016 | 1.22 (0.66 to 2.25) | 0.552 | 0.75 (0.36 to 1.55) | 0.432 |
| 2017 | 1.28 (0.71 to 2.31) | 0.408 | 0.94 (0.47 to 1.88) | 0.871 |
| 2018 | 1.90 (1.09 to 3.33) | 0.024 | 1.02 (0.51 to 2.00) | 0.964 |
| 2019 | 2.13 (1.21 to 3.75) | 0.009 | 1.29 (0.66 to 2.54) | 0.455 |
| OR = Odds ratio. ISS = Injury Severity Score. Missing Civil Registration number in 2010 (n = 4), 2011 (n = 16), 2012 (n = 19), 2013 (n = 4), 2014 (n =11), 2015 (n = 12), 2016 (n = 6), 2017 (n = 12), 2018 (n =18), 2019 (n = 11). | | | | |

Table S5: Catchment area and admission type for trauma patients from 2015–2019.

|  | 2015, n (%) | 2016, n (%) | 2017, n (%) | 2018, n (%) | 2019, n (%) |  |
| --- | --- | --- | --- | --- | --- | --- |
| Catchment area based on location of injury |  | | | | |  |
| Aarhus Municipality | 215 (56.6) | 237 (50.6) | 287 (50.3) | 265 (49.1) | 222 (48.8) |  |
| Surrounding municipalities | 165 (43.4) | 231 (49.4) | 284 (49.7) | 275 (50.9) | 233 (51.2) |  |
| Aarhus Municipality with ISS > 15 | 37 (34.6) | 42 (32.3) | 48 (30.1) | 49 (29.9) | 31 (22.0) |  |
| Surrounding municipalities with ISS > 15 | 70 (65.4) | 88 (67.7) | 107 (69.0) | 115 (70.1) | 110 (78.0) |  |
| Transferred patients | 53 (11.3) | 59 (11.1) | 51 (8.5) | 45 (8.1) | 36 (7.6) |  |
| Transferred patients with ISS > 15 | 38 (27.5) | 33 (22.3) | 35 (21.5) | 26 (15.4) | 26 (17.7) |  |
| Helicopter | 48 (10.3) | 54 (10.2) | 66 (11.0) | 62 (11.1) | 63 (13.4) |  |
| ISS = Injury Severity Score. Missing catchment area data in 2015 (n = 88), 2016 (n = 64), 2017 (n = 30), 2018 (n = 19), 2019 (n = 17). Missing data for Helicopter admission in 2015 (n = 3) and 2019 (n = 1). | | | | | |  |
